# Airway management following head and neck microvascular reconstruction: is tracheostomy mandatory?

**DOI:** 10.1016/j.bjorl.2021.07.007

**Published:** 2021-10-13

**Authors:** Ory Madgar, Nir Livneh, Alex Dobriyan, Elad Dagan, Eran E. Alon

**Affiliations:** a Sheba Medical Center, Department of Otolaryngology and Head and Neck Surgery, Tel Hashomer, Israel; b Tel Aviv University, Sackler Faculty of Medicine, Israel; c Sheba Medical Center, Department of Oral and Maxillofacial Surgery, Tel Hashomer, Israel

**Keywords:** Microvascular reconstruction, Free flap, Tracheostomy, Head and neck

## Abstract

**Objectives:**

Airway management following maxillofacial microvascular reconstruction is a fundamental part of the perioperative management. In oral cavity microvascular reconstruction, the airway is potentially compromised by airway edema, flap edema or bulkiness, hematoma formation, or upper airway sequelae from surgery. Classical teaching advocates elective tracheostomy in patients undergoing maxillofacial free flap reconstruction, while others keep patients intubated overnight (delayed extubation). The optimal method for perioperative airway management is still debated. This study aim was to evaluate the morbidity associated with elective tracheostomy in patients undergoing maxillofacial microvascular reconstruction and to recognize those patients who can avoid elective tracheostomy.

**Methods:**

Retrospective review of patients who underwent maxillofacial microvascular free flap reconstruction, between November 1^st^ 2010 and October 31^st^ 2019 in our center.

**Results:**

One-hundred and nine patients underwent microvascular reconstruction in the maxillofacial region. Sixty-one patients underwent an elective tracheostomy upon the primary surgery. Forty-eight patients were left intubated overnight. Seven patients underwent a late tracheostomy which was performed upon a neck re-exploration for postoperative complications, except for one patient which was due to failed extubation attempt. Patients who didn’t receive an elective tracheostomy were younger and had a shorter duration of postoperative hospitalization. Seven patients suffered from tracheostomy- related complications, all of them underwent elective tracheostomy and none were from the late tracheostomy group.

**Conclusions:**

Our results suggest, the routine use of elective tracheostomy in maxillofacial microvascular free flap reconstruction is unnecessary. Elective tracheostomy should be considered on case-to-case basis.

**Level of evidence:**

4.

## Highlights

• Airway management following maxillofacial microvascular reconstruction is complex.
• Lack of consensus exists regarding the optimal airway management perioperatively.
• Routine use of tracheostomy is unnecessary.
• Tracheostomy should be considered on a case-to-case basis.

## Introduction

Surgical resection with or without immediate reconstruction is one of the main treatment modalities for head and neck malignancies.^1^ Microsurgical free flap reconstruction is used widely in day-to-day practice as part of the reconstruction elevator after resection of head and neck tumors.^2^, ^3^, ^4^ Depending on the site, size and complexity of the defect, the reconstruction may involve free tissue graft of soft tissue, bone or both.^1^ Airway management following maxillofacial head and neck microvascular reconstruction is a fundamental part of the perioperative management of these patients.^5^, ^6^ In oral cavity microvascular reconstruction the airway is potentially compromised by airway edema, flap edema or bulky flap, hematoma formation, upper airway sequelae from the surgery, as well as comorbidities related to tobacco use, which is prevalent in this population.^7^ Classical teaching advocates elective tracheostomy in patients undergoing maxillofacial head and neck free flap reconstruction, while newer trends advocate keeping patients intubated overnight (delayed extubation). The optimal method for perioperative airway management is still debated,^6^ and the decision is often dependent on surgeon’s training and experience. In a national postal survey sent to maxillofacial surgical units in the United Kingdom, 69% of the units (39/57) perform elective tracheostomy “almost always” or “usually” following free flap reconstruction in the head and neck.^1^

Although tracheostomy provides a safe airway it has been identified as an independent predictor for postsurgical complications.^6^ The elective tracheostomy complications rate is between 4%–45% according to the literature including bleeding, cannula obstruction or displacement, airway obstruction, fistula formation, and pneumonia. In addition it can prolong patients’ postoperative hospitalization.^6^, ^8^

Recent work by Rogers et al. evaluating patients’ experience of tracheostomy following head and neck microvascular reconstruction demonstrated that 60% of patients would “very much” choose to avoid tracheostomy if at all possible.^9^ Furthermore, there has been a recent trend towards delayed extubation following maxillofacial free flap reconstruction.^7^, ^8^ We retrospectively reviewed our patients who underwent free flap reconstruction in the maxillofacial head and neck region to evaluate the morbidity associated with elective tracheostomy in these patients and to recognize the group of patients in which elective tracheostomy could be spared.

## Methods

This is a retrospective analysis of medical charts of all patients who underwent microvascular free flap reconstruction in the maxillofacial head and neck region involving the oral cavity, from 1^st^ of November 2010 to 31^st^ of October 2019 at our department.

Most surgical procedures were performed with a two-team approach, including head and neck, maxillofacial and reconstructive surgeons. Tracheostomy was usually performed at the beginning of surgery, in cases where there was a question about the necessity of a tracheostomy the patient was evaluated at the end of surgery. Depending on flap size, bulkiness, location, airway involvement, and patient’s comorbidities a decision to perform tracheostomy was made. Patients who did not undergo tracheostomy were left intubated overnight, the next day the patients were evaluated and usually underwent extubation. All patients were left overnight at the postoperative anesthesia recovery unit, extubation was usually performed over a tube exchanger following a positive leak test, and patients were monitored for signs of respiratory distress for several hours in the recovery unit following extubation. Afterwards, patients were transferred to the relevant inpatient unit.

Study protocol was approved by institutional Ethics committee (number: 4635-11-SMC).

## Results

During the study period 129 patients underwent microvascular free flap reconstruction in the head and neck region at our department. One hundred and nine patients underwent free flap reconstruction in the maxillofacial head and neck region (Table 1) involving the oral cavity. The vast majority of cases included primary resection and immediate reconstruction of malignant tumors; there were a few patients with benign diseases who underwent primary resection and immediate reconstruction, and a few secondary reconstructions. The group consisted of 56 patients with oral cavity reconstructions (including mandible), 31 tongue reconstructions and 22 maxilla reconstructions. In total there were 52 radial forearm free flaps, 37 fibula free flaps, 17 anterolateral thigh free flaps, two scapula free flaps and one iliac crest free flap. Patient’s average age was 59.7, there were 53 males and 56 females. Sixty-one patients underwent an elective tracheostomy upon the primary surgery. Forty-eight did not undergo an elective tracheostomy and were left intubated overnight. Seven patients out of the 48 ended up undergoing a late tracheostomy, and only one of these were due to a failed extubation attempt on the first postoperative day. The other six patients underwent a late tracheostomy during a neck exploration for postoperative complications, and not due to respiratory distress.

**Table 1:** Comparison between patients who underwent elective tracheostomy to those who were left intubated overnight in the all study population.

		Elective tracheostomy	No elective tracheostomy			
			Total	No tracheostomy	Late tracheostomy	
**Number**		61	48	41	7	
**Age (years)**		62.1*	56.7*	56.2	59.7	*p = 0.036
**Gender**	Males	30	23	21	2	
	Females	31	25	20	5	
**BMI**		24.94*	26.34*	26.5	25.2	*p = 0.04
**Post-op duration (days)**		17.2*	13.06*	11.2	24.1	*p = 0.027
**Flap type**	RFFF	20	32	28	4	
	FFF	23	14	11	3	
	ALT	17	0	0	0	
	Scapula	1	1	1	0	
	Iliac crest	0	1	1	0	
**Site**	Tongue	21	10	8	2	
	Oral cavity	31	25	21	4	
	Maxilla	9	13	12	1	
**Neck dissection**	Total	47	30	24	6	
	Unilateral	35	25	21	4	
	Bilateral	12	5	3	2	
**2^nd^ operation**		18 (29.5%)	7 (14.5%)	1	6	

Non-significant differences were left blank.

BMI, Body mass index; RFFF, Radial Forearm Free Flap; FFF, Fibula Free Flap; ALT, Anterolateral thigh.

* Marks statistically significant difference, p value shown.

As presented in Table 1 patients who did not receive an elective tracheostomy were younger, had a higher BMI and a shorter duration of post-operative hospitalization (these differences were statistically significant).

We performed a univariate analysis to assess for predisposing factors for avoiding tracheostomy, as seen in Table 1 younger patients and those with higher BMI were more likely to avoid tracheostomy. Other factors such as resection site, flap type, performance of neck dissection, and gender were not significant. Upon multivariate analysis there were no statistically significant factors.

Seven patients (6.4%) suffered from tracheostomy- related complications; all of them underwent an elective tracheostomy (Table 2). One patient suffered from tracheal stenosis which was treated by a bronchoscopy and balloon dilatation. Another patient suffered from supra-stomal stenosis and was also operated on. Two patients suffered from stomal bleeding. Two patients underwent early accidental decannulation, which for one of these patients led to prolonged resuscitation and surgical revision of the stoma. This patient suffered eventually from flap failure requiring a second flap. One patient suffered from a stomal infection that was treated conservatively. 63 patients (57.8%) who underwent tracheostomy were decannulated, for 58 of them decannulation was performed during the hospitalization (Table 2). Out of our cohort four patients suffered from pneumonia, they underwent an elective tracheostomy. The average duration until decannulation was 19.4 days for patients who underwent an elective tracheostomy and 15.67 days for the patient who underwent late tracheostomy (*p* = 0.38). Twenty-five patients underwent a second operation, 18 of them were from the elective tracheostomy group, and seven patients did not undergo a tracheostomy in the primary surgery. Out of the seven patients who did not undergo an elective tracheostomy six underwent a late tracheostomy during the second operation and one patient did not; three of the patients who underwent a late tracheostomy ended up undergoing another surgery during the following 30 days.

**Table 2:** Tracheostomy related complications and decannulation rates for all patients who underwent tracheostomy.

	Elective tracheostomy (n = 61)	Late tracheostomy (n = 7)
**Number of patients with tracheostomy related complications**	7	0
**Number of patients suffering from post-operative pneumonia**	4	0
**Number of patients decannulated**	57 (93.4%)	6 (85.7%)
**Number of patients decannulated during hospitalization**	52 (85.2%)	6 (85.7%)
**Average number of days to decannulation**	19.4	15.67

## Discussion

There is a lack of consensus regarding whether or not a tracheostomy is needed following microvascular reconstruction in the maxillofacial-head and neck region.^5^, ^7^, ^8^, ^10^^,^^11^ The decision is often dictated by the surgeon’s training, experience, postoperative care, and beliefs and is not evidence based. Different medical centers and even different surgeons within the same center may differ in their philosophy regarding the use of routine elective tracheostomy.^1^, ^8^ The use of tracheostomy to secure the airway for maxillofacial free flap reconstruction continues to be widespread despite the complications related to its use, the lack of evidence regarding its necessity, and the fact that patients generally fear it.^9^, ^11^ The goal in the perioperative period following maxillofacial head and neck microvascular reconstructions is to provide a safe transitional airway to protect against surgery-induced transient alterations of the upper aerodigestive tract.^11^ The ideal airway should have no long-term morbidity, aid return to normal physiological functions particularly speech and swallowing, and enable early discharge from the hospital without increasing readmission rates. Tracheostomy has several distinct advantages in this setting, including a safe airway, the potential for early discontinuation of mechanical ventilation, decreased sedation requirements, ease of access for respiratory care, decreased dead space, decreased airway resistance, and improved pulmonary mechanics. Tracheostomy also provides a safe and fast airway in case of a need for a flap take back to the operating room, in these situations’ reintubation might be difficult and time consuming thus it may prolong flap ischemia time. Furthermore, the flap itself could be injured during the intubation. However, despite its advantages, tracheostomy is a surgical procedure with appreciable morbidity.^11^

A recent study by Rogers et al. evaluated patients’ experience with temporary tracheostomy following head and neck microvascular reconstruction. Generally, patients reported a negative experience; a majority of patients stated that they would “very much” avoid a tracheostomy if possible. The main problems concerned fear and communication. One-third to one-half of patients stated that they had had “very much” or “quite a bit” of a problem in regard to choking, discomfort, attracting attention, sleeping, and general management (other than the suctioning).^9^

Following Rogers et al. study we performed a retrospective review of all patients who underwent microvascular free flap reconstruction in the head and neck region, in aim to improve our patients overall experience, evaluate tracheostomy related morbidity in these patients and safety of avoiding elective tracheostomy.

Patients who did not undergo an elective tracheostomy were generally younger and had a shorter duration of postoperative hospital stay and higher BMI. The difference in hospitalization duration could be explained by the duration of the decanulation process or need to equip the patient’s home before being discharged with a tracheostomy. Similar differences in hospitalization duration were seen in other series.^11^, ^12^ The lower BMI rates in the tracheostomy group might be due to malnutrition related to advanced disease which required a bigger resection which led to tracheostomy. The difference in age might be explained by fewer pulmonary diseases and better pulmonary reserves. For those patients who required re-intubation due to respiratory distress or re- exploration there were no intubation- related complications.

Patients who underwent late tracheostomy did not suffer from any stoma-related complications and were not decannulated later than those who underwent elective tracheostomy in our series. Two thirds of patients who were left intubated overnight were reconstructed using a radial forearm free flap. Avoiding elective tracheostomy was possible for oral cavity, tongue, and maxilla reconstruction groups.

Despite the goal of providing a safe airway there were more airway complications and pneumonias in patients who underwent an elective tracheostomy. In fact, none of the patients who underwent delayed extubation suffered from pneumonia, and only one patient had an airway- related complication of failed extubation which required performing a tracheostomy. On the other end in the tracheostomy group 4 cases of airway complication, one of which even led to prolonged resuscitation and eventually flap failure requiring a second flap.

Although upon univariate and multivariate analysis performance of neck dissection was not found to be a statistically significant predisposing factor for elective tracheostomy performance or avoidance, there were only five patients who underwent bilateral neck dissection in the group of patients who did not undergo elective tracheostomy (Table 1). Out of these five patients two patients ended up undergoing a late tracheostomy, and one of these two patients needed a tracheostomy due to a failed extubation attempt on the first postoperative day. This was the only patient in our cohort who failed his extubation attempt. These finding, although not statistically significant, raises the question whether avoiding an elective tracheostomy in patients undergoing free flap reconstruction and bilateral neck dissection is safe, as this group of patients are at higher risk for respiratory compromise. This issue should be further studied as there three patients in our cohort who underwent free flap reconstruction and bilateral neck dissection and did not necessitate a tracheostomy.

**Figure 1:**
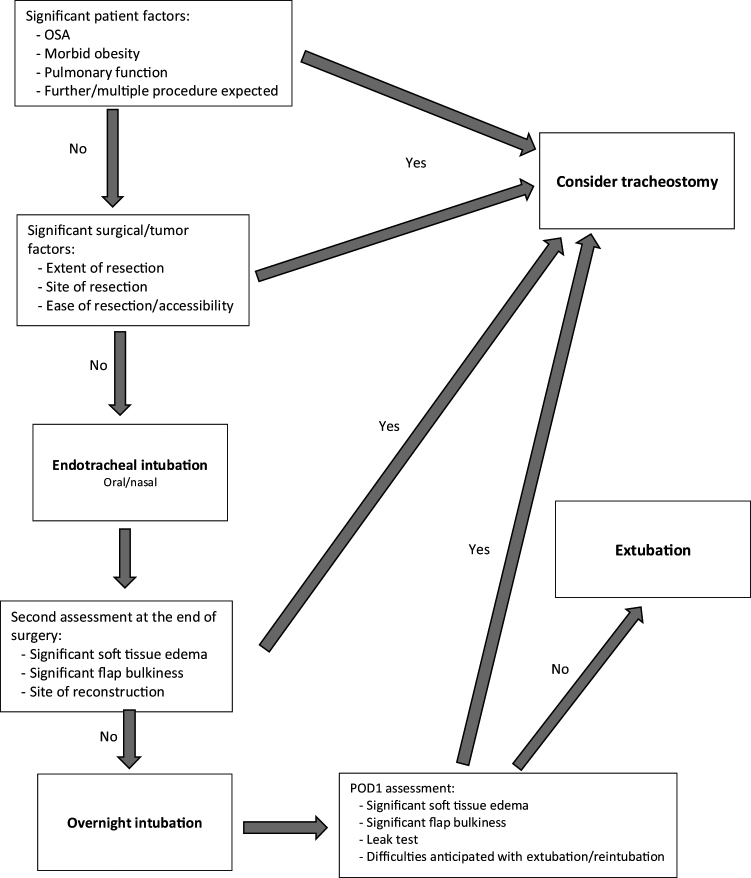
Suggested algorithm for decision making in regard to the perioperative airway management following maxillofacial-head and neck microvascular reconstruction.

Our results suggests that overnight intubation is a safe option and can be performed instead of an elective tracheostomy for patients undergoing maxillofacial head and neck region microvascular reconstruction. Proper patient selection is fundamental, as our results suggests that for younger patients avoiding an elective tracheostomy can be considered. Although not statistically significant our clinical experience and results suggest that patients with low bulk soft tissue volume free flap are good candidates to avoid tracheostomy. Particularly those who require minor reconstruction such as anterior lateral tongue or anterior oral cavity with no need for base of tongue reconstruction, or palate. Patients’ comorbidities and specific flap locations should be taken into consideration. Although subjective, it is important to assess the status of the oropharyngeal airway at the completion of the operation. If there is any concern for postoperative oropharyngeal airway obstruction because of edema or packing, one should perform a tracheostomy. Furthermore, patients that have a more than usual risk for a return to the operating room should be considered tracheostomy tube candidates as well. Attached in Fig. 1 is our suggested algorithm for decision making in regard to the perioperative airway management following maxillofacial-head and neck microvascular reconstruction.

Further studies are needed to define an objective selection criterion for aid in the airway management following microvascular reconstruction in the oral cavity.

## Conclusions

The routine use of elective tracheostomy in the maxillofacial head and neck region microvascular free flap reconstruction is unnecessary. As our results suggest decision regarding elective tracheostomy should be considered on a case-to-case basis. Tracheostomy can be avoided, particularly in younger patients with low bulk flaps having minor reconstruction of the anterior lateral tongue or anterior oral cavity with no need for base of tongue reconstruction, or of the palate. If there is any concern at the end of the operation for postoperative airway safety tracheostomy should be performed.

## Funding

This research did not receive any specific grant from funding agencies in the public, commercial, or not-for-profit sectors.

## Conflicts of interest

The authors declare no conflicts of interest.
